# Strategies to Promote Health Equity in Interventional Cardiology: Practice, Research, Education, and Policy

**DOI:** 10.1016/j.jscai.2026.104267

**Published:** 2026-03-17

**Authors:** Robert M. Califf

**Affiliations:** Division of Cardiology, Department of Medicine, Duke University School of Medicine, Durham, North Carolina

**Keywords:** health equity, interventional cardiology, policy, research

This issue of *JSCAI* includes a panoply of articles examining various areas pertinent to health equity in interventional cardiology. This represents a timely focus in the face of growing evidence that disparities in access to clinical care and variations in clinical outcomes as a function of social drivers are continuing to evolve in adverse directions. At the same time, the concept of health equity itself is under fire, with shifts in criteria for government funding of related research, including direct opposition from high levels in government to research and actions addressing health equity.

The concept of health equity is rooted in the belief that everyone deserves a fair opportunity to achieve their highest level of health, regardless of background or circumstances. Furthermore, the fundamental foundation for biomedical ethics rests on 4 key principles: autonomy, beneficence, nonmalfeasance, and justice.^1^ In this context, justice is generally thought of as ensuring fairness in treatment and resource allocation, while beneficence means doing good for the patient, and nonmalfeasance means avoiding harm. Addressing the complexity of our fiduciary duty to the patient’s well-being while also respecting individual autonomy is central to the ethical practice of medicine.

Accordingly, efforts to understand and repair inequity should be integral to public health and medical care. Efforts to address factors affecting longevity and functional status of individuals and populations, regardless of root cause, are driven by identifying the causes of poor outcomes and then developing and implementing interventions and policies aimed at improving them, followed by empirical assessment of what works and what does not. When the cause is a biological factor, such as older age, or a measurable biological process, such as coronary thrombosis or valvular immobility, we develop drug, device, and behavioral interventions to improve outcomes. Similarly, when outcomes differ as a function of social or behavioral factors, systemic barriers—such as poverty or discrimination, or lack of access to good jobs, quality education, housing, and high-quality health care—they must also be addressed. Here, the essential role of individual health systems and clinicians is to better understand and avoid bias when making clinical decisions and allocating effort and resources.

When seeking to identify and correct health inequities in the field of interventional cardiology, it may be useful to consider 3 phases. First, is there inequity in access to interventional cardiologists and facilities? Second, are there differences in the outcomes of procedures? And third, are there differences in postprocedural care and outcomes (an important aspect that has got less attention than it deserves)? Although larger issues of overall health outcomes and disparities beyond the field of interventional cardiology are important and fascinating, they are too large to address in this editorial comment.

## Who gets access to interventional cardiology expertise and services?

The specific question of whether access to appropriate interventional cardiology expertise services is fair and reasonable brings into play basic factors of wealth and geography. Considerable existing evidence,^2^ further reinforced by investigations in this issue of *JSCAI*, shows that age, sex, race, wealth, education, and place of residence (rural vs urban) all influence access to the interventional cardiologist. Inequities in access will be difficult to address completely until a more rational system for allocating health services is adopted. In a society where longevity and functional status are directly related to wealth, and people with a college education outlive those without by an average of 8 years, access to interventional cardiology clinical care is not the most significant factor in play, but it seems like a problem that could be addressed. The issues arising from rural vs urban residence are also addressable but require policy changes in addition to personal efforts.

## Among those who get access, are there inequities in outcomes?

The overall outcome of an interventional cardiovascular procedure depends on factors including patient characteristics, the facility and personnel involved in the procedure, and access to and utilization of appropriate secondary preventive care. The evidence for disparities once a patient gains appropriate access to the expertise and facilities in interventional cardiology is clear.^3^ There is strong evidence that women and racial minorities in general have more procedural complications. There is also evidence (as in most medical procedures)^4^ that given the same clinical scenario, factors such as race, sex, and socioeconomic status can affect recommendations for who undergoes the procedure.^5^ A particular measure, door-to-balloon time,^6^ offers metrics that highlight significant issues about how to directly address clinical decision-making and engineer the process of care by the cardiovascular care team.^7^

## Postprocedure care

There is increasing recognition that an interventional procedure constitutes a critical landmark for initiating critical postprocedure care that includes vigilance for complications and prevention of recurrent events or disease progression. Yet disparities persist in terms of concordance with guideline-directed care after interventional procedures, just as they do in noninterventional clinical care.^8^ Additionally, disparities in referral to cardiac rehabilitation programs are well documented.^9^

## Global differences

The lens of geographic differences, from national to more local scales, is critical for understanding equity. The majority of people live in low- and middle-income countries with limited capacity for interventional cardiology. Furthermore, with increasing wealth and successful public health measures, such countries have experienced dramatic reductions in infant mortality and death from infectious diseases, so that chronic diseases (and specifically cardiovascular disease) are the largest and fastest-growing causes of death and disability. Stark differences in access to care across countries and within developing economies raise uncomfortable challenges. Several articles in this issue make the point that we can measure inequities across the world and systematically work to reduce them.

## Clinicians and the systems in which they work

Articles in this issue have appropriately interpreted clinical workforce composition as an important issue in health equity. The primary goal should be to have (1) the most technically competent interventional teams, (2) operating in adequate facilities that are (3) accessible to patients who (4) meet an evidence-based indication for a procedure that (5) can reduce the risk of death and improve functional status. A team that is reflective of the population is a secondary goal; however, there is evidence of patients deriving real benefit in the primary care setting from seeing clinicians who “look like them.”^10^ For reasons that have been analyzed in detail, interventional cardiology lags most other medical specialties in representation of women and racial and ethnic minorities.

## Clinical trials

To the extent that access to clinical trial participation is regarded as a benefit, there is ample and growing literature to indicate that inequities are present. Furthermore, because the purpose of a clinical trial is to estimate the effect of an intervention on a population, there is reason to worry that the extrapolation could be flawed if trial participants do not reflect the population to whom the results will be extrapolated. Recent data suggest that clinical trials in interventional cardiology are characterized by an underrepresentation of women, racial minorities, and people who live in rural areas; however, approaches to correcting this problem are being developed.^11^

## Three instructive examples

A concerted effort to reduce disparities in ST-elevation myocardial infarction provides one potential pathway for improving health equity in interventional cardiology. The sentinel finding of ST-segment elevation, as identifying patients whose survival is directly related to the time required to reperfuse the infarct-related artery, enabled systematic activation of a cascade of events to get the patient’s occluded artery open as quickly as possible. Regardless of who the person is, the system is intended to act the same way, including by bypassing facilities, using air transport, and other methods to reduce door-to-needle time. High-quality registries are in place to monitor performance, and when issues with access and outcomes are found, performance improvement strategies are implemented. Although the system is not perfect, the drive to get this right at the individual, regional, national, and global levels provides a great example of what can be done to improve care for all.

The issue of valvular heart disease provides another example. Given the evolution of valve replacement and repair to include percutaneous and less risky surgical approaches, it is difficult to justify the complexity of the current diagnostic and referral process. Systems are being implemented to use machine learning to automate the continuum of the electrocardiogram, echocardiogram, and consultation for consideration of a procedure.^12^ The ubiquity of ECG and echo technologies opens the door to systematic availability of procedures proven to yield improvements in survival and quality of life.

Congenital heart disease presents a more complex situation but may offer a useful opportunity to correct inequities with major consequences for better clinical outcomes. Approximately 1% of infants are born with congenital heart disease. The ubiquity of echocardiography has produced a digital record of all of these cases, and there are fixed and well-characterized programs with various levels of facilities and expertise. The outcomes are measurable, and the potential impact of a rational referral system has been modeled.^13^

## Summary

Clinical outcomes for a person for whom evidence supports referral to an interventional cardiovascular specialist and a subsequent procedure vary as a function of disease severity, comorbidities, and social drivers of health. There are many simple analogies in life, such as sports, in which a rational approach to improving performance and outcomes is to use specific interventions to correct deficiencies that are measurable to close the gap in performance. The first step is to seek agreement on the principle that equity is a basic goal of health care. As I have noted, we too often take this principle for granted when, in fact, our policies are not aligned with this goal. For interventional cardiology as a discipline and for the individual cardiologist, it is important to try to provide equitable care to patients at the clinic or interventional facility, but this is not enough. In addition to delivering equitable care before, during, and after the procedure, equitable access is an important goal worthy of individual and policy-oriented analysis and intervention. Including workforce and global considerations in the effort seems reasonable, particularly through a long-term lens. But there is also plenty of “blocking and tackling” work to do in the short term on the basics of access, engineering the best team effort for procedures, and improving postprocedure care in the practice of cardiovascular medicine.

## Declaration of competing interest

The author declared no potential conflicts of interest with respect to the research, authorship, and/or publication of this article.
